# Chemical components of tattoo inks and their potential role in carcinogenesis and their side effects

**DOI:** 10.1007/s00204-026-04484-y

**Published:** 2026-06-27

**Authors:** Luiza Czaczkowska, Ewa Jabłońska, Julia Bury, Natalia Szurant, Wioletta Ratajczak-Wrona

**Affiliations:** 1 Department of Immunology, Medical University of Bialystok, 15-269, Białystok, Poland; 2 Poland Białystok,

**Keywords:** Biological reviews, Carcinogens, Tattoos, Inflammation

## Abstract

**Abstract:**

Tattooing is an invasive procedure that has gained popularity, particularly among individuals. Growing attention has been directed toward the safety of tattoo ink ingredients, which are complex mixtures of synthesis precursors and byproducts. These inks may contain classified carcinogens, heavy metals, polycyclic aromatic hydrocarbons (PAHs), and phthalates, often at concentrations exceeding permissible limits. Tattooing involves the insertion of needles 1–3 mm into the dermis. While part of the pigment is eliminated with epidermal turnover, approximately 25% is transported to lymph nodes and subsequently into the bloodstream. The remaining pigment is engulfed by phagocytic cells involved in innate immunity. Repeated needle punctures trigger local inflammation, recruitment of immune cells, and release of inflammatory mediators, followed by tissue regeneration and remodelling driven by keratinocytes, fibroblasts, and endothelial cells. Cases of malignant melanoma, squamous cell carcinoma, keratoacanthoma, and lymphoma have been reported in individuals with tattoos. However, the relationship between tattooing and cancer is not fully understood. It is suspected that the cause may be the transfer of toxic substances to lymph nodes and then to organs. The possible carcinogenic effects of tattoo ink ingredients will be discussed based on available scientific reports.

**Graphical Abstract:**

**Figure:**
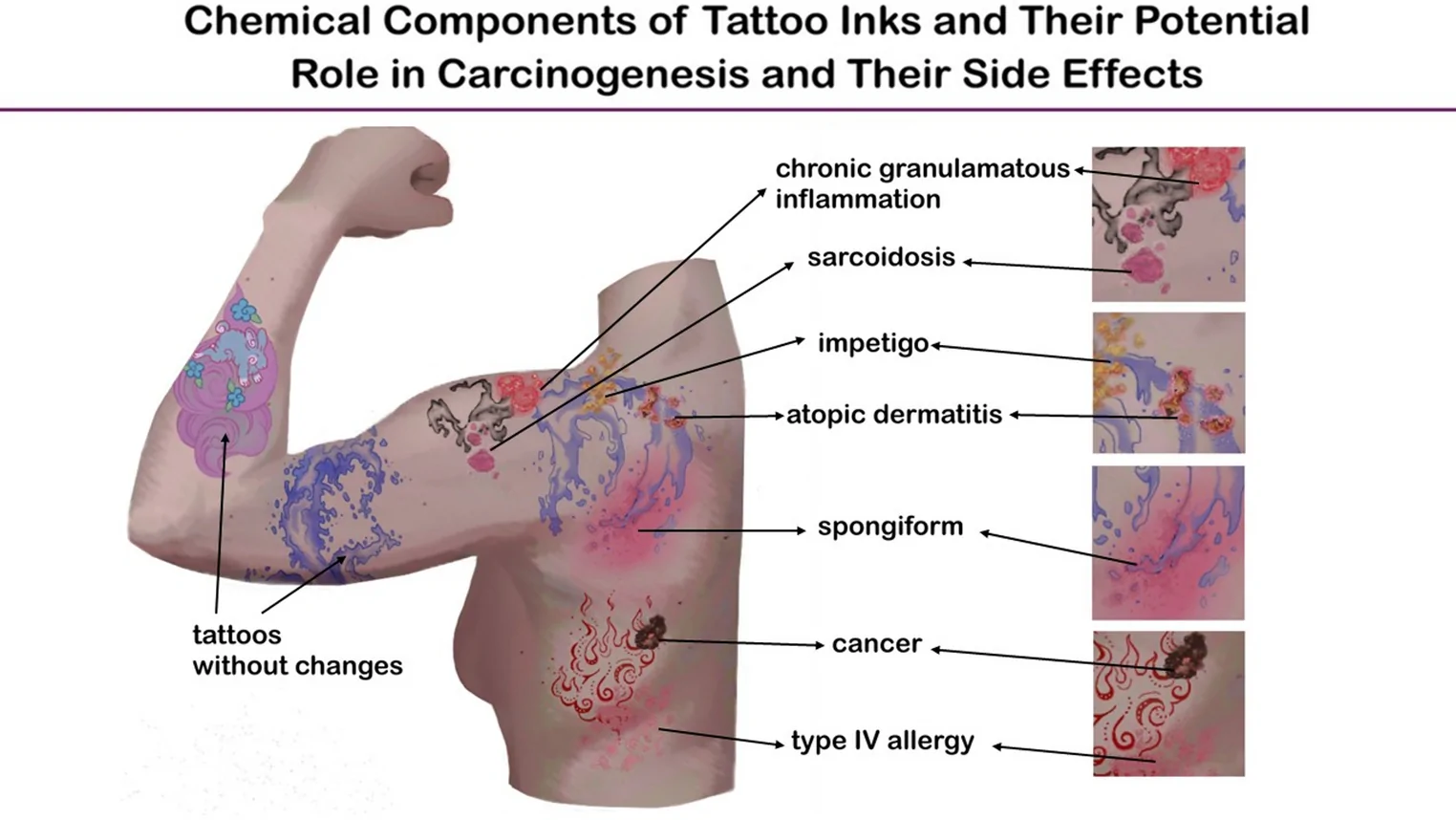

## Introduction

The largest organ in the body, the skin, covers the entire external surface. It also has structures that allow it to perform specific functions. These include hair follicles, sweat glands that help regulate body temperature, and sebaceous glands that secrete sebum, which lubricates the skin and hair, preventing these structures from drying out. It serves as the body’s first and protective barrier against pathogens, ultraviolet light, chemicals, and mechanical damage. The skin is composed of three main layers. The epidermis is the layer that contacts the external environment. Beneath it lays the dermis, and even lower lays the hypodermis. Each of these layers is differentiated depending on its function and location in the body (Dodig et al. 2024; Lotfollahi 2024; Yousef et al. 2025).

Tattooing is an invasive procedure, especially popular among young adults and adults below 40 years old. The highest prevalence is recorded in the United States of America, but lately it has also become popular in the European Union. Unfortunately, the safety of tattoo ink’s ingredients is questioned. Many research reports skin inflammations, infections, allergic reactions, autoimmune diseases, and cancers, due to pigment components. Tattoo ink is a mixture of precursors and by-products of synthesis. The skin consists of epidermis, dermis, and adipose tissue. The tattooing process involves inserting a needle to a depth of approximately 1–3 mm into the dermis layer. The pigment is shed along with the epidermis, but approximately 25% of the ink injected into the skin reaches the lymph nodes and from there into the bloodstream. The portion remaining in the skin comes into contact with immune cells, including phagocytes, which are a key element of the nonspecific immune response (Dodig et al. 2024).

The constant punctures of the needle during tattooing create inflammation. Primary immune cells are recruited at this site, and inflammatory mediators are released, preventing the spread of pathogens that may have entered the skin during puncture. Macrophages and neutrophils absorb the pigment, and as the cells disintegrate, the released ink is removed, but in small quantities; most remain in the skin. In addition to macrophages, neutrophils, and dendritic cells, connective tissue fibroblasts also absorb the pigment at the site of inflammation. Langerhans cells are a highly specialized type of dendritic cell found in the skin. They also play a significant role in the ink removal process. They are responsible for transporting the ink to the lymph nodes. Thanks to the growth of keratinocytes, fibroblasts, and endothelial cells, the process of epidermal reconstruction and remodelling occurs. The process of rebuilding the damaged tissue takes about a year. After this time, the tattoo becomes less intense. This is because the visible ink is a remnant retained in the dermis by immune system cells (Dodig et al. 2024).

There have been reports of malignant melanoma, squamous cell carcinoma, keratoacanthoma, and lymphoma in patients with tattoos. However, the possible links between tattooing and malignant tumors are not fully understood. It can be speculated that they may be caused by the movement of toxic substances. This topic is hotly debated in numerous scientific reports in immunology and toxicology. Tattoo ink contains heavy metals such as nickel and chromium, polycyclic aromatic hydrocarbons (PAHs), and phtalates, which are part of Endocrine Disrupting Chemicals (EDCs). Their permissible concentration in the ink are exceeded (Brusasco et al. 2025; Dodig et al. 2024; Hosseini et al. 2025; Kolena et al. 2025; Lebhar et al. 2024).

Current scientific reports do not confirm mutagenic effects of tattoo inks; they only indicate allergic reactions, granulomatous reactions, and various skin diseases. The compounds whose metabolites were detected in urine were phthalates, both high molecular weight (HMWP) and low molecular weight (LMWP). The article we reviewed showed high urinary concentrations of both phthalates in the first three days after tattooing, with higher concentrations observed in larger tattoos, consistent with the amount of ink used in the process. Furthermore, HMWPs, such as DEHP, undergo additional oxidative processes, resulting in a longer half-life of these compounds and exposure to their metabolites (Kolena et al. 2025).

## Materials and methods

The study conducted research on the PubMed and Google Scholar Platforms. The articles used in our study were meta-analyses, cohort studies, epidemiological studies, integrated bioinformatic analysis, research articles, in vitro studies, and case–control studies with relevance for tattoo-related cancers. We also searched for information on the ingredients contained in tattoo ink and the legal regulations related to it. The legal acts and regulations in this article were researched on a website dedicated to European Union legal regulations.

Accessed articles were published between 2015 and 2025, with the majority published in the last five years. Among the searched phrases on the PubMed platform, “tattoo-related cancer” had 114 entries, “tattoo” had 3,191 results, and “tattoo ingredients” had 52 results. Excluded articles did not contain enough content or research data to support their claims. Excluded articles also did not add new specific information.

## Results

Tattoo inks are primarily composed of pigments and colourants, which are responsible for visual effects. They are suspended in solvents and supplemented by preservatives, thickeners, and binders. Most substances in tattoo inks are organic components. Inorganic compounds, including iron oxide, titanium dioxide, and chromium oxide, can also be found in them. High concentrations of metal nanoparticles (NPs) such as chromium, copper, lead, mercury, and zinc are commonly found in tattoo inks. Binding agents provide stability, solvents aid in ink flow, and additives, thixotropic agents, and surfactants improve performance and protect against contamination. (“ < em > IARC Monographs < /em > hazard classification,” n.d.; Battistini et al. 2025; Charuta et al. 2023; Peebles et al. 2025).

Green dyes may contain chromium, nickel, arsenic, aluminium and copper; blue dyes may contain chromium, nickel, copper, and cobalt; yellow dyes may contain cobalt, lead chromate, diarylide pigments, and cadmium; red dyes may contain lead, antimony, arsenic, beryllium, nickel, and mercury; black dyes beside Carbon Black, contains beryllium, iron oxide, arsenic, chromium, cobalt, lead, nickel; white ink contains titanium dioxide, zinc oxide, barium sulfate. Some of these compounds are also classified by the International Agency for Research on Cancer (IARC) as carcinogenic or potentially carcinogenic. Benzo[a]pyrene, which is a polycyclic aromatic hydrocarbons (PAHs), and is classified as a Group 1 carcinogen, exists in carbon black ink, and is produced through the incomplete combustion of petroleum products. Despite the ban on the use of some of these compounds in Europe and their absence on labels, their presence has been confirmed. Furthermore, these dyes are not specifically regulated or tested for products intended for subcutaneous injection, unlike pharmaceuticals (Charuta et al. 2023; Kiszla et al. 2023; Peebles et al. 2025).

Many of the substances mentioned above have already been categorized by IARC. The classification is presented in Table 1. Group 1 includes compounds designated as carcinogens. These include carbon black, cadmium sulfate, beryllium, arsenic, mercury sulfate, lead, and cadmium compounds. Group 2A, or possibly carcinogenic, includes lead chromate, chromium, cobalt, and nickel. Group 2B, containing possibly carcinogenic compounds with low evidence, includes titanium dioxide, diarylide pigments, cobalt aluminate, and nickel (Peebles et al. 2025).

**Table 1:** Representation of carcinogenic substances found in tattoo inks

Pigment	Yellow/ Orange	Black	White		Green		Blue	Red
Possible contaminants	Diarylide pigments	Carbon Black	Titanium dioxide		Chromium		Chromium	Antimony
	Lead chromate	Nickel	Barium sulphate		Nickel		Nickel	Nickel
	Cobalt	Cobalt	Zinc oxide		Arsenic		Cobalt	Arsenic
	Cadmium	Arsenic			Copper		Copper	Mercury
		Beryllium			Aluminium			Beryllium
		Lead						Lead
		Iron oxide						
		Chromium						
Carcinogenic classification	Group 1			Group 2 A		Group 2B		
	Carbon black Cadmium sulfate Beryllium Arsenic Mercury sulfate Lead Cadmium compounds			Lead chromate Chromium Cobalt Nickel		Titanium dioxide Diarylide pigments Cobalt aluminate Nickel		

Possible carcinogenic contaminants found in yellow/ orange, black, white, green, blue, and red dyes. Carcinogenic classification of IARC, where Group 1is carcinogenic to humans, Group 2 A is probably carcinogenic to humans, and Group 2B is possibly carcinogenic to humans. Group 3, which is not classifiable as to its carcinogenicity to humans, was not presented in the table

On the 14th December 2020, Commission Regulation (EU) of the European Parliament and of the Council concerning the Registration, Evaluation, Authorisation and Restriction of Chemicals (REACH) introduced new safety standards for substances or mixtures used for tattooing purposes. For chromium, the maximum concentration (by weight) was 0.00005%, for mercury 0.00005%, for copper 0.025%, for lead 0.00007%, for benzo[a]pyrene 0,0000005%, and for zinc 0.2%. These regulations were established for exposure to toxic substances delivered orally, so these data are not commensurate with the actual exposure through injection into the dermal. It should also be mentioned that the composition of inks, even those produced by manufacturers with high standards, is variable and may be potentially allergenic and toxic (Peebles et al. 2025; “Regulation - 2020/2081—EN—EUR-Lex,” n.d.; Serup 2023; Vitipon et al. 2025).

Laser tattoo removal procedures have also become increasingly common. The laser can cause chemical degradation of ink molecules, creating compounds that were not present in the original ink composition or were present in small amounts. As the author suggests, this topic is very poorly understood and deserves separate research. He also describes the appearance of volatile degradation products such as benzene, toluene, and styrene after irradiation of yellow pigments in the presence of TiO₂. Furthermore, the risks of potential side effects caused not only by metabolites of the ink components but also by cytotoxic reactions induced by the laser itself also increase (Bolt and Hengstler 2025).

**Fig. 1:**
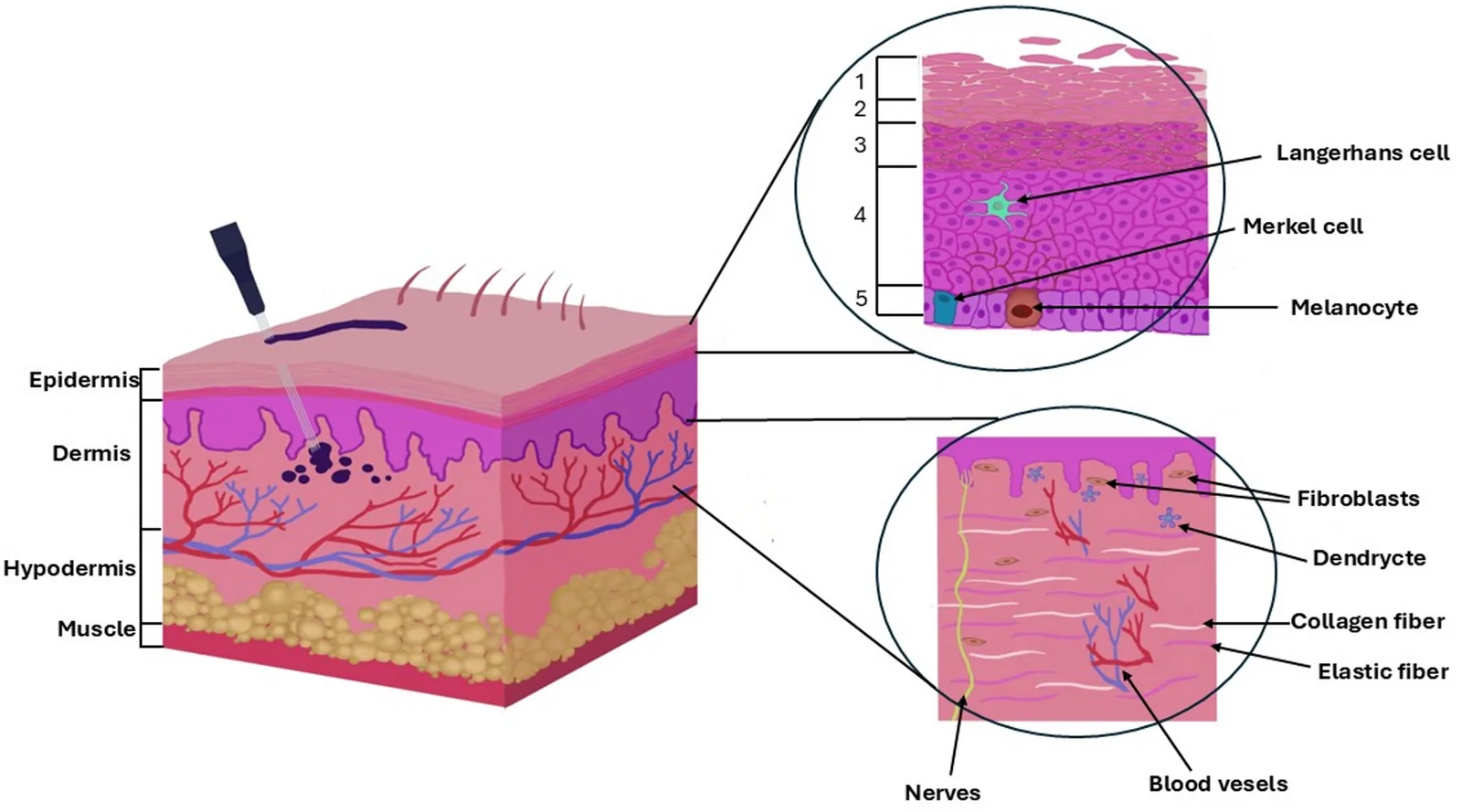
Skin structure-Visual representation of sublayers of Epidermis and Dermis. Epidermis layersare represented as punctuations from 1 to 5. 1-Stratum corneum, 2-Stratum lucidum, 3-Stratum Granulosum, 4- Stratum Spinosum, 5-Stratum Basale.

The skin consists of free main layers, and each one of them is divided into sublayers. This has been illustrated in detail in Fig. 1. Immunocompetent cells that are found in the layers of the skin plays important role in the process of skin renovation, after a tattooing procedure. The epidermis is divided into five layers: the stratum corneum, the lucid stratum, the granular stratum spinosum, and the basal stratum. The thickness of the epidermis is from 0.5 to 1.5 mm. It is differentiated into thin and thick epidermis. This layer contains no blood vessels. It consists of keratinized cells and stratified squamous cells, which form horny cells. Langerhans cells, keratinocytes, and Merkel cells are also present. Dendritic cells are present in the stratum spinosum. They play a very important role in the immune system. They are one of the cells that are capable of presenting antigens. Melanocytes are present in the Stratum basale and provide UV protection. Thin skin has four rows of epithelial layers, while thick skin has five layers (Dodig et al. 2024; Lotfollahi 2024; Yousef et al. 2025).

The majority of collagen found in the skin is located in the dermis. The percentage of collagen decreases with age, but it performs important biomechanical functions. This layer is composed of connective tissue, and its thickness varies depending on location. Its maximum thickness is 4 mm on the fingers. Connective tissue connects the dermis to the subcutaneous tissue and supports the epidermis. The surface of this layer is irregular, with numerous epidermal ridges. It strengthens the dermoepidermal junction exposed to mechanical stress. This structure also contains blood vessels and nerves that nourish the epidermis. The dermis is divided into a superficial papillary area and a deep reticular area. The former consists of an extracellular matrix and fibroblasts, which secrete fibronectin and hyaluronic acid. It also contains blood and lymphatic vessels, nerves, and epithelial cells. The thin papillary layer consists of loose connective tissue with type I and III collagen fibres, where mast cells, dendritic cells, and leukocytes are also located. This part of the layer penetrates the basal lamina through collagen VII fibrils, connecting the dermis to the epidermis. The deep reticular dermis is thick and woven from dense fibroelastic connective tissue, which also contains an extensive network of vessels, type I collagen fibres, and fibroblasts. The presence of elastin fibres gives the skin its elasticity (Abdallah et al. 2017; Dodig et al. 2024; Lotfollahi 2024; Mescher 2024; Zwirner and Hammer 2024).

The subcutaneous layer, also called hypodermis, lays under the dermis. It is composed of vascularized, loose connective tissue that connects the skin to organs and allows for gliding over them. The extensive blood vessels present here allow for the rapid absorption of medications administered via injection directly into the adipose tissue. This layer, composed primarily of multiple layers of adipocytes, serves as a cushion and insulator for internal organs and stores fat (Lotfollahi 2024; Mescher 2024).

Side effects of tattooing consisted of acute side effects such as: skin irritation, hypersensitivity to bacterial and viral infections. Long-term effects found were as follows: chronic inflammation, autoimmune diseases, atopic dermatitis, local or systemic sarcoidosis, delayed type IV allergy, UV-induced cancer, lymphoma, multiple myeloma, basal cell carcinoma, and bladder cancer.

## Discussion

### Immunology of the skin and lymph nodes

The skin, the most external organ in the human body, is exposed to various factors. The body’s defences include simple and advanced mechanisms of the immune system, which include: proper pH, the epidermal barrier, adequate skin hydration, proinflammatory cytokines produced by keratinocytes, and the highly specialized lymphatic tissue of the skin. Since 2011, the skin has been referred to as a peripheral lymphatic organ. Skin-associated lymphoid tissue (SALT) is part of the skin immune system (SIS), which is located in the epidermis and dermis. The most important functions of this system are performed by immunocompetent cells located in these layers. These include Langerhans cells (LC), melanocytes, keratinocytes, mast cells, subpopulations of dendritic cells (DC), macrophages, and T lymphocytes.

#### Immunocompetent cells

##### Keratinocytes

Keratinocytes (KC) participate in innate and adaptive immunity and perform many important functions in the skin’s immune response. They are the main source of antimicrobial peptides (AMPs). Reduced or increased expression of AMPs is observed in various skin diseases and in susceptibility to bacterial infections. Keratinocytes that produce AMPs participate in promoting angiogenesis and wound healing. One such peptide is LL-37, which increases in response to injury. In the case of skin injury, TLRs present on the surface of keratinocytes are activated. Interferons (IFNs) are produced, and through the inflammasome signaling pathway, proinflammatory cytokines are produced. KC inflammatory responses may be triggered by release from ruptured cells, detection of pathogen-associated molecular patterns (PAMPs), or detection of signals from other cells in the skin. IL-1 increases the expression of intercellular adhesion molecule (ICAM)-1 on dermal endothelial cells, LC cells, and MHC class II. This allows leukocytes to migrate more easily to the skin at the site of inflammation. In addition to proinflammatory cytokines such as IL-1, keratinocytes produce IL-6, IL-10, and TNF. Due to their ability to produce chemokines and their receptors, and to express chemokine ligands such as CXCL1, CXCL9, CXCL10, CXCL11, and CCL20, keratinocytes can express MHC class II and function as antigen-presenting cells (APCs) for T lymphocytes when stimulated with IFN-γ. They can also stimulate CD4 + and CD8 + lymphocytes (Matejuk 2018; Simmons and Gallo 2024).

##### Langerhans cells

Langerhans cells are transformed macrophages found in the epidermis and considered the guardians of skin immunity. They also act as antigen-presenting cells (APC) and can migrate to lymph nodes. They present a single peptide via MHC molecules to naive T lymphocytes in the lymph nodes or to memory T lymphocytes in the skin. They can also activate lymphocytes to perform adaptor functions, including Th1, Th2, and Th17 responses, as well as the suppressor response of regulatory T lymphocytes. LCs possess differentiation antigens. They are identified by CD1a, HLA-DR, and CD207 (langerin). Each differentiation antigen serves a distinct function. CD1a presents lipid antigens of microorganisms to T lymphocytes; HLA-DR, as an MHC class II receptor, serves as a ligand for T lymphocyte receptors, and the langerin receptor mediates endocytosis of pathogens. CD207 forms Birbeck granules (BCs), which are specific organelles for LCs. In addition to these cells, which are characteristic of them, they can be expressed on some cells of the dermis. Therefore, LCs are considered a subset of dendritic cells (DCs) (Chen et al. 2025; Neagu et al. 2022).

Langerhans cells were divided into 4 subsets by sequencing: CD207 + CD1a + LC1 in the steady state with the ability to self-renew, CD207loCD1c + CD1b + LC2 with increased migration capacity but reduced viability, and CD207loCD1aloCCR7 + LC corresponding to activated, migrating LC. Flow cytometry, in turn, previously divided them into two main subpopulations: LC1, which are specialized in cytokine secretion and participate in the innate immune response, and LC2, which mediate the adaptive and tolerogenic response (Chen et al. 2025; Neagu et al. 2022).

Langerhans cells, which participate in wound healing, are recruited to the site of inflammation thanks to their ability to migrate. Keratinocytes continuously communicate with Langerhans cells, which, thanks to their expression of Major histocompatibility complex class I-related chain B (MICA B) and lymphocyte activation receptor natural killer group 2D (NKG2D ligand), enable them to migrate into the wound environment. For LCs to repopulate the epidermis, circulating monocytes are recruited through the expression of the chemokine receptors CCR-2 and CCR-6. Immature LCs expressing langerin, CD24, and EpCAM require cooperation with IL-34 and TGF-β secreted by keratinocytes to begin to perform their fully functional functions. During the inflammatory phase of tissue remodelling, LCs repopulate the epidermis thanks to TNF-α, which increases the expression of surface adhesion molecules (Chen et al. 2025).

##### Melanocytes

Melanocytes participate in the immune response, including through melanization, which facilitates the phagocytosis of pathogens and participates in the formation of reactive oxygen species (ROS). Melanosomes are modified lysosomal structures that contain numerous enzymes, such as α-mannosidase, acid phosphatase, β-N-acetylglucosaminidase, β-galactosidase, and acid lipase. They are susceptible to oxidative stress. During melanogenesis, these cells produce a significant amount of free radicals and hydrogen peroxide. Under cellular stress, melanocytes produce heat shock proteins (HSPs) and NKG2D ligands, the expression of which exposes melanocytes to immune- targeted destruction. Pattern recognition receptors (PRRs) allow these cells to recognize pathogens and initiate the production of type 1 IFNs, proinflammatory cytokines, and chemokines (Speeckaert et al. 2022).

##### Dendritic cells

There are two types of DC, one of which lays in the epidermis and the other in the dermis of the skin. Dermis DCs (dDCs) express epithelial-cell adhesion molecule IL-10, and have the ability to stimulate the transformation of lymphocytes B into plasma cells secreting IgM antibodies. The two main subtypes of dermal DCs are langerin- and CD103 + -expressing cells. These cells participate in planned reactions thanks to their organization of a network of cytokines and chemokines. Tissue macrophages and dendritic cells share common precursors. The dDC subpopulation, which is a macrophage-like cell, expresses factor XIIIa and CD136, which may be a key aspect of wound healing (Matejuk 2018).

The homeostasis of SALT depends on the functioning, integrity, and elasticity of lymph vessels and nodes, as well as the smooth interaction of immune cells with the skin environment. SIS is also closely linked to the microflora found on the skin surface. Resident and transient microflora develop an immune response. The former is acquired during birth, when it is passed from mother to child. The transient microflora is acquired through contact with the external environment, and the composition of both the resident and primary microflora stabilizes in adulthood and is unique to each individual (Abdallah et al. 2017; Gołąb et al. 2023; Matejuk 2018).

The immune system is divided into innate (primary) and adaptive (secondary). Direct activation and a rapid response against pathogens are provided by the innate immune system. It consists of DCs, NK cells, and macrophages, which possess pattern-recognition receptors (PRRs) on their surface, including Toll-like receptors (TLRs) and C-type lectin receptors. Alongside these, there are also NOD-like receptors (TLRs), which recognize pathogen-associated molecular patterns (PAMPs) and other microbial structures, including peptidoglycan. This is achieved through the activation of nuclear factor kappa B (NF-κB) and mitogen-activated protein kinase (MAPK) pathways. The inflammatory environment stimulates cells of the innate immune system to secrete more inflammatory mediators (Abdallah et al. 2017; Gołąb et al. 2023).

When skin is damaged, or pathogens are recognized via TLRs, keratinocytes produce proinflammatory cytokines, including IL-1β, IL-33, tumor necrosis factor (TNF-α), which induce E- and P-selectin, and intercellular adhesion molecule 1 (ICAM-1), initiating leukocyte adhesion to the endothelium and subsequent diapedesis. The first components of the immune response to appear at the site of inflammation are neutrophils and monocytes, which have the ability to phagocytose. They will secrete further inflammatory mediators, resulting in the recruitment of more cells. The secondary immune response, involving lymphocytes, develops somewhat later. Proinflammatory cytokines produced by keratinocytes will also activate DCs, which are pattern-recognition cells, and leukocytes originally located in the skin (Abdallah et al. 2017; Gołąb et al. 2023; Jiang et al. 2020).

T lymphocytes, which participate in adaptive immunology, mature in primary lymphoid organs and then migrate via the bloodstream to secondary lymphoid organs, where, upon detecting a specific antigen, they transform into effector T lymphocytes. This gives them the ability to migrate to various organs, including the skin. Lymphoid cells (LCs) are capable of antigen presentation, and some exhibit a high cutaneous tropism. Innate lymphoid cells (ILCs) comprise five subgroups: NK cells, ILC1, ILC2, ILC3, and lymphoid tissue-inducing cells. ILC1s produce IFNγ and TNF, ILC2s produce IL-4, IL-5, IL-9, and IL-13, and ILC3s produce IL-17, IL-22, and GM-CSF. They are present in the dermis and interact with mast cells. When an inflammatory response occurs in the skin, effector T cells are recruited. Following their activation and differentiation in secondary lymphoid organs, expression of CD62L and CCR7 is reduced, and expression of specific molecules is increased in the peripheral tissues from which the injury originated. In lymph nodes draining the skin, expression of CD62P, CD62E, CCR4, and CCR10 is increased. Expression of CCL17 and CCL27 on epidermal keratinocytes facilitates the recruitment of CCR4 + T cells and CCR10 + T cells to the skin (Egawa and Kabashima 2020; Meininger et al. 2020; Tikoo et al. 2018).

### Immune’s reaction to tattooing

Tattooing, an invasive procedure, is preceded by an immediate, acute inflammatory phase, followed by a proliferation and maturation phase to restore tissue integrity and the epidermal renewal process. During the healing of tattoo wounds, a reduced production of collagen fibers has been observed. This is a rather grim outlook, as collagen is responsible for skin elasticity, and the ink particles, by acting on the precursor of type I collagen protein, lead to a reduction in its number (Falconi et al. 2009).

After tattooing, three main cells participate in internalizing the ink in the skin: neutrophils, macrophages, and fibroblasts. Due to the mechanical trauma caused by the disruption of the skin barrier through continuous punctures of the dermis, neutrophils are the first to reach the site of inflammation and participate in a nonspecific cellular response. After approximately 24 h, they are eliminated by macrophages, which, through continuous cycles of ink capture, release, and re-capture, ensure the lifelong visibility of the ink. Fibroblasts capture a small portion of the pigment compared to macrophages, which are constantly replaced by new cells that transform from monocytes. An imbalance between the inflammatory and regenerative responses of tissues, and between pro-inflammatory M1 macrophages and anti-inflammatory M2 macrophages, leads to a disruption of the adaptive immune response, chronic inflammation, and autoimmune diseases (Devcic et al. 2022).

The ingredients of the inks vary in size. The organic part consists of nanoparticles. The rest is much larger. In a study by Lin et al. Nanoparticle migration to lymph nodes has been observed, and from there, they can be transported through the bloodstream to other internal organs. Larger particles, due to their size, cannot be transported there and likely migrate to the liver. Importantly, the size of the inks used for medical procedures is constant and cannot be varied. Additionally, they are coated with substances that reduce their uptake by immunocompetent cells and nonspecific adsorption. Unfortunately, the same cannot be said for inks used for tattooing. Increasing their stability would likely lead to reduced absorption by macrophages, and consequently, uneven pigmentation, which, for aesthetic reasons, professionals want to avoid at all costs (Lin et al. 2024; Signe Bedsted Clemmensen et al., 2025).

Cobalt and zinc, found in a wide range of pigments, are essential for the functioning of the human body. Cobalt is, among other things, a cofactor for vitamin B12. However, when absorbed in high doses, it can be toxic and has a harmful effect on fibroblasts, leukocytes, and macrophages. The gradual release of this chemical element by macrophages can lead to altered function and carcinogenic potential. Zinc participates in many enzymatic reactions, vitamin A metabolism, and immunoregulation, but zinc oxide nanoparticles released in large quantities affect the immune system, particularly macrophages. Devcic et al. correctly noted that macrophages exposed to these compounds exhibited a more pro-inflammatory phenotype and decreased their effectiveness in fighting pathogens and cancer cells. Moreover, the authors also report a lower effectiveness of macrophages in tissue regeneration. This is argued to be due to the removal of monocytes, which were treated as a by-product, by immune surveillance mechanisms, which may be an additional argument for the occurrence of autoimmune diseases (Devcic et al. 2022).

### Permanent makeup and medical tattoos

Permanent makeup, which also involves tattooing the skin, differs from regular tattooing by a shallower needle injection depth and the use of different inks. Pigments are based on iron oxide and can take on various shades. Fe2O3 has a red tint and is very stable, Fe3O4 is black and stable, and FeOOH is yellow and unstable. Various combinations of these pigments are used in permanent makeup to match skin tone. In patients with iron deficiency, percutaneous absorption of iron may occur, so when applying this makeup, it is important to consider preventive or additional supplementation of this element. Medical tattooing, such as scar camouflage, nipple-areola tattooing, or colon mapping and oncological radiotherapy (to visualize the location of the area to be treated), is treated more stringently than regular tattoo inks. Manufacturers are required to comply with chemical safety standards (Devcic et al. 2022; Serup 2023; Vitipon et al. 2025).

Jin and Chang analyzed taupe yellow ink and red wine ink pigments, in which they found inorganic substances. Substances added to red wine-colored ink were titanium (Ti), aluminum (Al), sulfur (S), chlorine (Cl), and iron (Fe). Detected in the taupe yellow ink pigment were iron (Fe), titanium (Ti), sulfur (S), and aluminum (Al) (Jin and Chang 2022).

Colored pigments with physical particles, in most tattooing cases, will remain in the tattoo site. Whether it is possible to perform tattooing depends not only on the capabilities of the tattooed person’s body, but also on the composition of the ink. Due to the elements provided for tattooing, certain controversies have arisen, and the inability to perform the investigated operations that are exposed to these risks, such as lymph nodes, and above all, protective devices, research on ten topics through many protective devices (Serup 2023).

### Side effects of tattooing

While the short-term effects of tattoos are well known, their long-term effects are rarely discussed. They can cause autoimmune diseases, spongiform or atopic dermatitis, impetigo, and local or systemic sarcoidosis. Depending on the inks used and their ingredients, other reactions can be expected. Red inks can cause delayed type IV allergy and may also increase the risk of UV-induced cancer. Black inks can cause chronic granulomatous inflammation and sarcoidosis. Along with blue pigment, they have been linked to various cases of skin cancer (Ranalli et al. 2024).

The carcinogenic mechanism and granuloma formation may be linked to an accelerated immune response by macrophages and other immune system cells. Inks that do not contain toxic substances specified in REACH do not lead to an accelerated immune response. Inks containing heavy metals, among other substances, led to a stronger immune response (Lin et al. 2024).

Larger tattoos deliver a higher dose of ink, and therefore, also of undesirable compounds. This results in longer exposure time and a higher dose of potentially toxic substances. When injected into the skin, tattoo ink causes severe inflammation, which can lead to chronic inflammation and an increased risk of abnormal cell proliferation, particularly lymphoma, multiple myeloma, and basal cell carcinoma. Furthermore, tattooed individuals have a higher risk of lymphoma (Nielsen et al. 2024; Signe Bedsted Clemmensen et al. 2025).

One of the largest cohort studies (CRABAT) comprised 13,239 tattooed and 101 184 non-tattooed participants. Exposure was measured by calculating total body tattoo coverage, taking into account the tattoo’s filler. Notes were also taken regarding tattoo colour, coverage area, post-treatment healing, and other environmental factors. It is clear that the results of such a large cohort study will contribute important information to the discussion (Hosseini et al. 2025).

Lymphadenitis associated with tattoos is common. Lymphadenopathy in individuals with a history of cancer is also a cancer risk factor. Foster et al. present the case of a patient whose primary tumor site, despite the diagnosis, was unknown. The patient was diagnosed with stage IV undifferentiated carcinoma. Due to multiple inconclusive biopsy results from various lymph node locations, the etiology of the cancer remained unknown for a long time. Meanwhile, the patient continued to notice lymph node swelling until biopsies of the left and right axillary lymph nodes revealed acute lymphadenitis with reactive follicular hyperplasia. Microscopic findings suggested an acute inflammation secondary to tattoo ink. The authors also noted a new tattoo in the patient, extending from one arm, traversing the upper and middle back to the opposite arm. This case clearly demonstrates why tattoos should also be considered a cancer risk factor. It also demonstrates that ink deposits in nearby lymph nodes, and that individuals susceptible to cancer are unaware of the risks associated with tattoos (Foster and Sokhn, 2023).

Pizarro-Mondesir et al. discuss the unusual case of a man with suspected tattoo-related PAH exposure to bladder cancer. It is worth noting that the patient was a non-smoker with no family history of cancer, who, in addition to tattooing, may have had a brief, two-year occupational exposure to these compounds. Compounds belonging to the polycyclic aromatic hydrocarbons group are present in some tattoo inks. The patient was diagnosed with de novo urothelial carcinoma. The suspicion that this cancer was linked to the compounds contained in the ink was raised due to the fact that the patient underwent approximately 100 h of tattooing, resulting in tattoos covering both arms, the left leg, the chest, and the abdomen. Furthermore, benzo[a]pyrene, the most well-known PAH compound, belongs to the group of endocrine-disrupting compounds (EDCs) and has many potential carcinogenic effects, including breast cancer, thyroid cancer, liver cancer, and lung cancer (Desnavailles et al. 2024; Pizarro-Mondesir et al. 2024).

### Tattoos versus vacinnes

After vaccination against severe acute respiratory syndrome coronavirus 2 (SARS-CoV-2), many concerns arose about the impact of tattoos on vaccines. Particularly, what effect might vaccine administration have on individuals with tattoos? Capucetti et al. demonstrated that the presence of ink crystals at the vaccine injection site can modulate the vaccine-specific immune response. In the case of black and red inks, they can enhance the vaccine response. This may contribute to improving the efficacy of inactivated vaccines. Furthermore, the authors demonstrated a negative effect of accumulated ink on spike protein expression and the humoral response. The presence of ink at the vaccination site may potentially affect antigen presentation, local inflammation, and the innate immune response (Capucetti et al.,^2025^).

Haller et al. described the case of a 39-year-old Spanish woman who, one month after receiving a second dose of the Moderna COVID-19 vaccine, noticed rashes on her eyebrows and the upper portion of her left arm, on three of her four tattoos. Interestingly, the rash did not appear on the entire black tattoo, but only on the colored tattoos. According to the Naranjo scale for adverse drug reactions, the patient’s probability of the vaccine triggering the granulomatous rash is possible. The triggering factor, although rare, may be an immunological phenomenon, in this case, COVID-19 vaccination. Moreover, cutaneous sarcoidosis, with which the patient was diagnosed, can appear in previously traumatized areas (Haller et al. 2023).

Kluger presented the case of a 63-year-old patient who underwent two lip micropigmentations in January and March 2021, using red wine-colored ink (BioTouch, USA). At the end of April 2021, the patient received the first dose of the SARS-CoV-2 vaccine (Comirnaty, Pfizer-BioNTech). A month later, she reported painful swelling of her lips, along with redness and tingling that affected her feeding. When she received a booster dose of the vaccine in mid-July 2021, she reported a rapid flare-up of symptoms. Five days after the third SARS-CoV-2 vaccination, she reported an exacerbation of symptoms. The authors hypothesized that the patient’s allergy was exacerbated by vaccinations (Kluger 2022).

## Conclusions

1. Regulations regarding permissible concentrations of ink ingredients are designed for oral administration, not for other pharmaceutical substances administered via injection, as is the case with other substances. This oversight poses a serious threat to human health, and the actual maximum concentrations of potentially carcinogenic substances should also be significantly reduced. Studies establishing maximum injectable doses should be conducted as soon as possible, and stricter controls on ingredients used in tattoo inks should be implemented.
2. Evidence for the direct carcinogenicity of inks is limited, despite the fact that many pigments are carcinogenic according to the International Agency for Research on Cancer (IARC). An increased risk of lymphoma and local malignancies is observed.
3. The key mechanism supporting the carcinogenicity of tattoo inks consists of chronic inflammation, toxin migration of the tattoo ink, and its accumulation in lymph nodes. Colored pigments and large tattoos pose the greatest risk because of the area they cover.
4. Tattoos should also be screened by doctors as part of a medical history. Such individuals should be classified as part of a risk group. The awareness of the cancer risks associated with tattooing among people who undergo this procedure is unknown. Individuals in at-risk groups should be made aware of the potential consequences.
5. When it comes to permanent makeup, it’s also important to monitor iron levels and supplement this macronutrient appropriately. Legal regulations regarding this type of makeup using inks, as well as medical tattooing, are much more restrictive compared to the regular tattooing process and the inks used by tattoo artists.
6. There are clinical cases in the literature of vaccine complications associated with tattoos or permanent tattoos. Therefore, further research on the impact of vaccines on the immune system of tattooed individuals is warranted.
7. To avoid the risks associated with tattooing, it’s important to carefully check the ingredients of tattoo inks or discontinue use altogether. However, it’s important to remember that regulations regarding their composition are imprecise, so what’s listed on the label may not always reflect their actual composition, and compounds that should not be present in inks are unfortunately still detected in them.

## Data Availability

No datasets were generated or analysed during the current study.
